# Modelling the MYC-driven normal-to-tumour switch in breast cancer

**DOI:** 10.1242/dmm.038083

**Published:** 2019-07-26

**Authors:** Corey Lourenco, Manpreet Kalkat, Kathleen E. Houlahan, Jason De Melo, Joseph Longo, Susan J. Done, Paul C. Boutros, Linda Z. Penn

**Affiliations:** 1 Princess Margaret Cancer Centre, University Health Network, 101 College St, Toronto, ON M5G 0A3, Canada; 2 Department of Medical Biophysics, University of Toronto, 101 College Street Suite 15-701, Toronto, ON M5G 1L7, Canada; 3 Ontario Institute for Cancer Research, 661 University Ave, Suite 510, Toronto, ON M5G 0A3, Canada

**Keywords:** MYC, Driver oncogene, PI3K, Breast cancer, Cancer model, Microenvironment

## Abstract

The potent MYC oncoprotein is deregulated in many human cancers, including breast carcinoma, and is associated with aggressive disease. To understand the mechanisms and vulnerabilities of MYC-driven breast cancer, we have generated an *in vivo* model that mimics human disease in response to MYC deregulation. MCF10A cells ectopically expressing a common breast cancer mutation in the phosphoinositide 3 kinase pathway (PIK3CA^H1047R^) led to the development of organised acinar structures in mice. Expressing both PIK3CA^H1047R^ and deregulated MYC led to the development of invasive ductal carcinoma. Therefore, the deregulation of MYC expression in this setting creates a MYC-dependent normal-to-tumour switch that can be measured *in vivo*. These MYC-driven tumours exhibit classic hallmarks of human breast cancer at both the pathological and molecular level. Moreover, tumour growth is dependent upon sustained deregulated MYC expression, further demonstrating addiction to this potent oncogene and regulator of gene transcription. We therefore provide a MYC-dependent model of breast cancer, which can be used to assay *in* *vivo* tumour signalling pathways, proliferation and transformation from normal breast acini to invasive breast carcinoma. We anticipate that this novel MYC-driven transformation model will be a useful research tool to better understand the oncogenic function of MYC and for the identification of therapeutic vulnerabilities.

## Abstract (precis)

**Summary:** We present a MYC-driven transformation model of breast cancer that recapitulates the disease *in vivo* and which can be used to identify MYC-dependent cancer vulnerabilities.

## INTRODUCTION

MYC deregulation occurs in the majority of human cancers and is therefore an ideal therapeutic target (Kalkat et al., 2017). Deregulation can be broadly defined as any genomic, epigenetic or signalling pathway aberration that results in sustained and often elevated MYC function. Targeting this potent oncoprotein using traditional approaches has been difficult as MYC is a transcription factor lacking enzymatic pockets. Alternative strategies are therefore under intense investigation, including approaches that target MYC-DNA interactions with small-protein inhibitors such as Omomyc or ME47 (Lustig et al., 2017; Soucek et al., 2013). In addition, identifying the unique vulnerabilities that arise in a MYC-driven tumour can reveal targetable synthetic lethal interactions (Horiuchi et al., 2016; Hsu et al., 2015; Sato et al., 2015; Toyoshima et al., 2012; Zhou et al., 2014). Thus, there is a need for a diverse set of MYC-driven models to identify and test new therapeutic strategies in a MYC-transformed setting.

Many approaches have been developed to model MYC in cancer, including cancer cell lines and genetically engineered mouse models (GEMMs). Rodent models include the widely used Rat-1A cell line, the Eμ-Myc transgenic model of B-cell lymphoma and the MMTV-MYC model of breast cancer (Sabò et al., 2014; Stewart et al., 1984; Stone et al., 1987; Strasser et al., 1990). Additionally, human MYC-dependent models, such as the Burkitt lymphoma cell-line model P493-6 with the ability to regulate MYC levels, have been used extensively to interrogate transcriptional regulation by MYC (Lin et al., 2012; Pajic et al., 2000; Schuhmacher et al., 1999). Moreover, the MycER chimeric protein system, which can be applied to cell-line and mouse models of many cancer types, has been used for studies of MYC in the cell cycle, genomic instability, transformation and synthetic lethal interactions (Eilers et al., 1989; Felsher and Bishop, 1999; Kessler et al., 2012; Liu et al., 2012; Pelengaris et al., 1999, 2001, 2004). These models have provided several new insights into MYC biology, suggesting that the further development of diverse models will also provide important contributions, particularly if they are complementary and obviate associated limitations with existing models. For instance, GEMMs are time-consuming to manipulate genetically, are resource-intensive and often do not accurately model human disease at the histological level. Specifically, when modelling human breast cancer, there are numerous differences between mouse and human mammary gland development and the surrounding stroma that need to be considered (McNally and Stein, 2017). In addition, most cancer cell line models do not accurately reflect human tumours *in vivo*, are derived from already fully transformed tumour tissue, and are not necessarily driven by or dependent on deregulated MYC. We have therefore set out to design a functionally complementary MYC-driven transformation model that meets several criteria: it is fully transformed in response to, and dependent upon, deregulated MYC; it recapitulates human disease at both the pathological and molecular level *in vivo*; and it is easy to manipulate genetically. These criteria outline a model that best reflects the complexity of human tumours *in vivo*, while ensuring that it is possible to identify MYC dependencies. To this end, we used a non-transformed parental cell (MCF10A) and developed an isogenic panel of cell lines that could meet these standards and model the transformation of breast cancer upon the introduction of deregulated MYC. We evaluate MYC dependency *in vivo* and characterise the histological and mRNA expression changes that occur in these MYC-driven tumours.

## RESULTS AND DISCUSSION

### *In vivo* transformation of MCF10A cells is dependent on active PI3K signalling, deregulated MYC and the presence of MYC Box II

We chose to model breast cancer as MYC is often deregulated and contributes to the progression of this disease (Singhi et al., 2011; Stratikopoulos et al., 2015). Recent large whole-genome sequencing studies of breast cancer patient tumours revealed that the most frequently mutated and amplified oncogenes in breast cancer are PIK3CA and MYC, respectively (Nik-Zainal et al., 2016). PIK3CA is mutated in approximately 30% of breast cancer patient tumours, whereas MYC deregulation, primarily scored through amplification, occurs in approximately 20% of breast cancers (Chen and Olopade, 2008; Nik-Zainal et al., 2016). To model breast cancer transformation, we sequentially introduced these commonly mutated oncogenes into MCF10As, an immortal, non-transformed breast cell line (Soule et al., 1990). MCF10As were chosen owing to their inherently stable genome, which ensures that transformation is a consequence of the introduction of ectopic oncogenes rather than an indirect consequence of genomic instability and the selection of a transformed clone. In addition, the MCF10As are a relevant model as the few mutations acquired during spontaneous immortalisation are prevalent in human breast cancer, including CDKN2A deletion (Kadota et al., 2010). Finally, despite harbouring a focal amplification at chromosome 8q24, total MYC levels remain highly regulated, decreasing rapidly in response to antiproliferative conditions; this observation is characteristic of normal MYC regulation in non-transformed cells (Kadota et al., 2010; Wasylishen et al., 2013). Thus, the MCF10A cells fulfilled our criteria of a parental breast cell system on which to base our model.

**Fig. 1.**
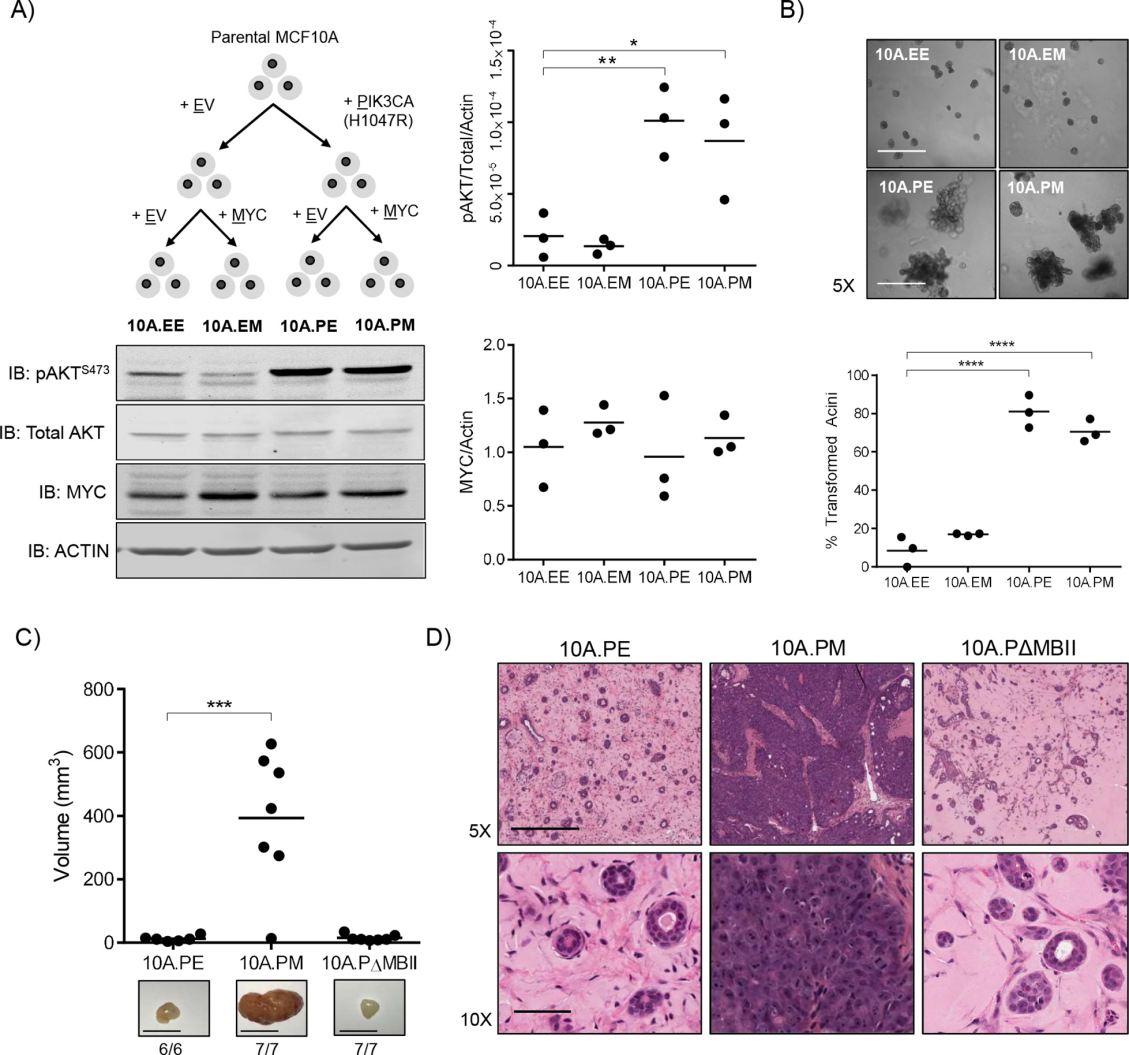
***In vivo* transformation of MCF10A cells is dependent on active PI3K signalling, deregulated MYC and the conserved MBII region.** (A) Schematic overview of the generation of an isogenic panel of MCF10A cell lines. Immunoblotting was performed against pAKT^S473^, total AKT, MYC and actin and quantified using ImageJ (*n*=3). One-way ANOVA with Bonferroni post-test for multiple testing. **P≤0.01, *P≤0.05. (B) The MCF10A isogenic panel cultured in Matrigel for 12 days. Images were acquired on day 12 and quantified based on an assessment of acinar circularity and size. Individual mean values from three biological replicates are shown; ****P≤0.0001, one-way ANOVA with Bonferroni post-test for multiple testing. Scale bars: 500 μm. (C) Individual tumour volume measurements from 10A.PE (*n*=6), 10A.PM (*n*=7) and 10A.PΔMBII (*n*=7) xenografts 49 days after injection. Representative images and take rate (mass formed/mouse injected) is indicated below each image. Scale bar: 0.5 cm. ***P≤0.001, one-way ANOVA with Bonferroni post-test for multiple testing. (D) Histology samples from xenografts in (C) stained with H&E. Representative images are shown. Scale bar for 5× images: 50 μm. Scale bar for 10× images: 500 μm.

We first stably introduced the empty vector control or the activated PIK3CA^H1047R^ allele into MCF10A cells. The latter was active in these cells as protein kinase B (AKT) phosphorylation at Ser473 was readily detectable (Fig. 1A). To this pair of cell lines, we introduced empty vector control or ectopic MYC expression, to model MYC deregulation through sustained expression as previously shown (Wasylishen et al., 2013). This resulted in a panel of four cell lines consisting of 10A.EE (empty vector, empty vector), 10A.EM (empty vector, MYC), 10A.PE (PIK3CA^H1047R^, empty vector) and 10A.PM (PIK3CA^H1047R^, MYC). Under growing conditions, we observed no significant change in either cell size or proliferation between any groups (Fig. S1A), suggesting that these cellular properties would not contribute to any phenotypes observed. Additionally, despite minor cell morphology differences (Fig. S1B), epithelial-to-mesenchymal transition (EMT) markers in the isogenic panel were not evident providing confidence that our cell-line panel had not undergone EMT transition, and thus represents a model of epithelial breast cancer (Fig. S1C).

To assay for transformation, we cultured our isogenic cell-line panel as breast organoids on Matrigel to model three-dimensional acini formation *in vitro* (Fig. 1B) (Debnath et al., 2003). The 10A.EE and 10A.EM cells formed round, organised acinar structures consistent with previous publications (Wasylishen et al., 2013). By contrast, 10A.PE and 10A.PM cells formed large and disorganised acinar structures, demonstrating a transformed phenotype driven by PIK3CA activation *in vitro*. MYC deregulation alone was insufficient to transform these acini, as previously described (Wasylishen et al., 2013), and did not potentiate the transformation of PI3KCA^H1047R^-expressing acini (Fig. 1B), suggesting that aberrant PI3K signalling was the main driver of transformation in this *in vitro* setting.

Despite no significant proliferative difference between these cell lines, activated PI3K signalling was sufficient for transformation in Matrigel. Considering the importance of environmental context on the phenotypes observed, we progressed to evaluate xenograft growth *in vivo*. Xenografts recapitulate the tumour microenvironment and offer an increasingly relevant context to study tumour formation and growth. MCF10A cells are non-transformed and are therefore not capable of forming xenografts in immunocompromised mice. In this manuscript, we refer to a xenograft as any human cell capable of engraftment in foreign species (mouse). We therefore assayed the isogenic panel developed in Figure 1A into female NOD-SCID mice (three mice per group) to observe if transformation had been achieved *in vivo* through expression of PIK3CA^H1047R^ and/or MYC (Fig. S1D)*.* As expected 10A.EE cells, containing no ectopic oncogenes, did not form any palpable masses *in vivo*, nor did 10A.EM cells. This was similar to our observation in organoid cultures showing that ectopic MYC alone was not sufficient for transformation. The most transformed organoid groups (10A.PE and 10A.PM) were able to establish palpable xenograft growths in all mice tested, suggesting that activated PI3K signalling is required for xenograft formation. Interestingly, despite no significant difference between the 10A.PE and 10A.PM cells in organoid transformation frequency, 10A.PM xenografts were much larger (Fig. S1D) and faster growing (data not shown) than the small, but palpable masses that arose from 10A.PE cells. To further interrogate the function of MYC in this assay, we generated cells expressing PIK3CA^H1047R^ and then introduced empty vector (10A.PE), MYC (10A.PM) or MYC with a deletion of MYC Box II (10A.PΔMBII) (Fig. S1E). The latter serves as a negative control, as MBII is evolutionarily conserved and required for MYC-dependent transformation (Oster et al., 2003; Stone et al., 1987). As observed previously (Fig. S1D), 10A.PE cells engrafted as small but measurable masses *in vivo* and 10A.PM cells were able to form significantly larger, and faster growing masses compared with 10A.PE cells (Fig. 1C). Cells expressing MYC^ΔMBII^ (10A.PΔMBII) formed small palpable masses, similar to 10A.PE, demonstrating that tumour xenograft growth was dependent on MYC and the conserved MBII region. Doubling times were 6 days for 10A.PM and 60 days for both 10A.PE and 10A.PΔMBII xenografts. Histological examination showed that 10A.PE and 10A.PΔMBII xenograft masses were composed of acinar structures embedded within an extracellular matrix, strikingly similar to the histology of normal human breast acini (Fig. 1D, Fig. S2A). By contrast, 10A.PM xenografts possessed major phenotypic differences, appearing as dense, fully transformed breast tumours with features similar to human disease. Given the role of PI3K signalling in the activation of prosurvival pathways (Manning and Toker, 2017), PIK3CA^H1047R^ enables MCF10A cells to establish small xenograft masses, yet does not provide the oncogenic activity to form fully transformed tumours. Although active PI3K signalling transforms breast organoids *in vitro*, both PIK3CA^H1047R^ and deregulated MYC are required for transformation *in vivo*.

### Deregulated MYC transforms breast acini into invasive ductal carcinoma *in vivo*

**Fig. 2.**
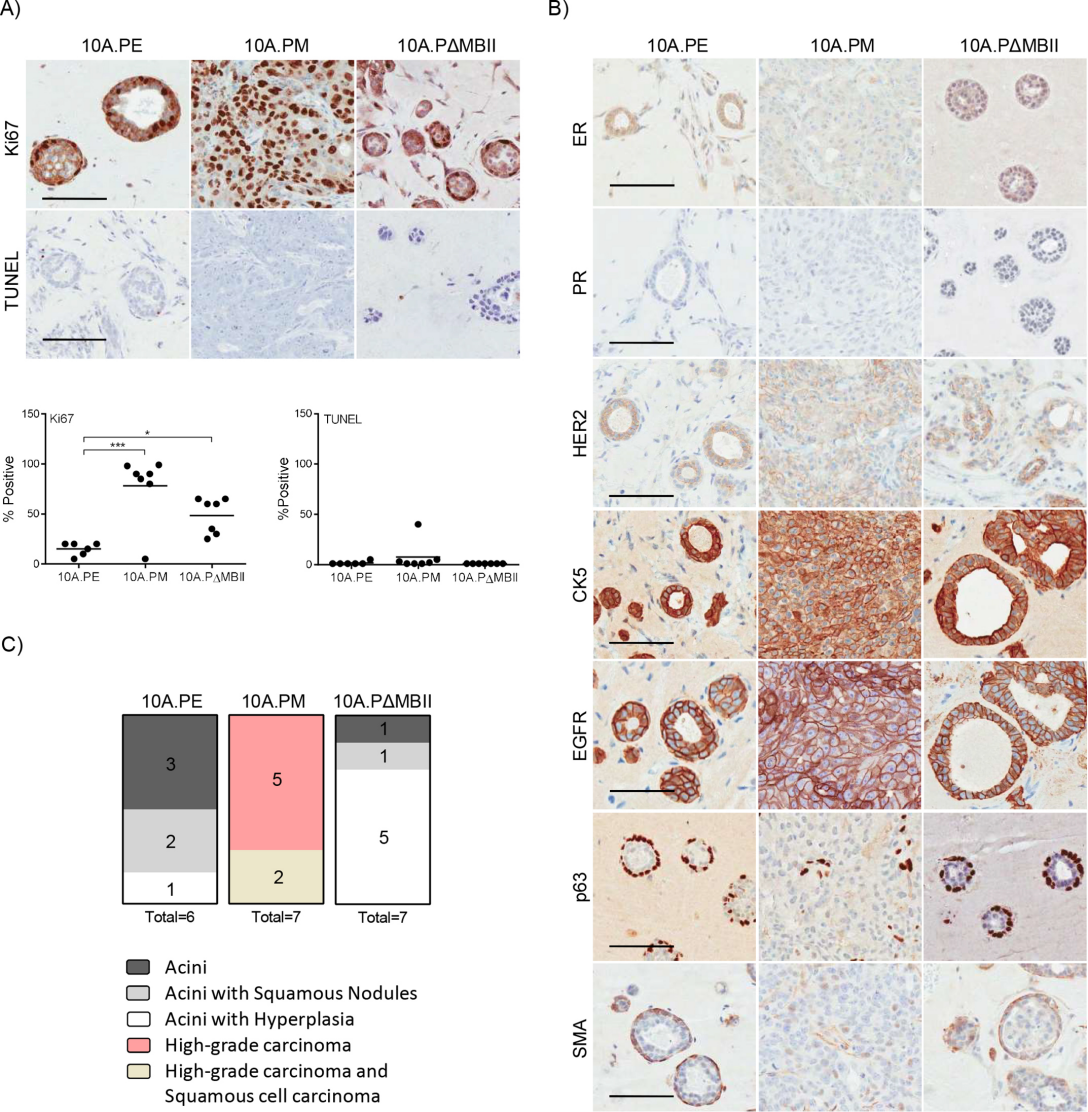
**Deregulated MYC transforms breast acini into invasive breast carcinoma *in vivo*.** (A) Tumours harvested in Figure 1C were stained (above) for Ki67 and TUNEL and were quantified (below) for the degree of Ki67 and TUNEL staining. Individual quantifications per tumour are shown; *P≤0.05, ***P≤0.001, one-way ANOVA with Bonferroni post-test for multiple testing. Scale bar: 100 μm. (B) Tumours harvested in Figure 1C were stained for ER, PR, HER2, CK5, EGFR, p63 and SMA by the Pathology Research Program Laboratory. (C) A pathology report was produced for each tumour using a combination of H&E, as shown in Figure 1D, and IHC markers used in this study. Scale bar: 100 μm.

In clinical practice, tumours are evaluated through histology, immunohistochemistry (IHC) and/or molecular profiling to characterise tumour subtype and features of aggressive disease. We applied these analyses to our xenografts, in a blinded-manner, with the assistance of practicing breast cancer pathologist Dr Susan Done. 10A.PM and 10A.PΔMBII growths had significantly increased Ki67 staining compared with 10A.PE, whereas TUNEL staining was low in all samples (Fig. 2A). Interestingly, although 10A.PΔMBII xenografts had an increase in Ki67 signal, the overall xenograft volume was equal to 10A.PE xenograft growths. This suggests that MYC^ΔMBII^ is able to confer some proliferative advantage owing to retention of partial MYC-related activity, but is not able to drive transformation; this observation is consistent with previous reports (Oster et al., 2003; Stone et al., 1987). We also analysed clinically relevant markers such as the estrogen and progesterone receptors (ER and PR), human epidermal growth factor receptor 2 (HER2) and cytokeratin 5 (CK5) as a marker of basal cells (Fig. 2B). The MCF10A cell line has been previously characterised as basal and triple-negative for breast cell receptors *in vitro* (Subik et al., 2010)*.* Introduction of PIK3CA^H1047R^ and MYC maintained the basal and triple-negative state *in vivo*.

We then used clinical markers to distinguish the degree of transformation within each xenograft with the assistance of Dr Susan Done. Malignant disease can be categorised as either carcinoma *in situ* (CIS) or invasive ductal carcinoma (IDC) and is distinguished by transformed cells contained within or invading through a layer of myoepithelial cells surrounding the acinar structure, respectively. To assess these conditions, we stained for myoepithelial cells using two markers, p63 and smooth muscle actin (SMA). 10A.PE and 10A.PΔMBII acini were surrounded by myoepithelial cells and were identified with human-specific epidermal growth factor receptor (EGFR) antibody, indicating that these cells were of MCF10A origin. In contrast, myoepithelial cells were not detected in 10A.PM tumours, as evidenced by a lack of p63 and SMA co-staining. Our pathology report revealed that a majority of 10A.PE growths were normal breast acinar structures displaying hollow lumen and a surrounding myoepithelial layer, with few cases of hyperplasia (Fig. 2B,C, Fig. S2B) (Dontu and Ince, 2015). The 10A.PΔMBII growths developed moderately more hyperplastic lesions than 10A.PE xenografts, despite MBII being essential for tumour growth. These data align with our Ki67 proliferation data and previously published results showing that although MBII is required for transformation, MYC^ΔMBII^ can retain some MYC-related activity in tissue culture assays (Oster et al., 2003; Stone et al., 1987). By contrast, 10A.PM tumours, which were highly proliferative and lacked myoepithelial cells, were scored as high-grade IDC. Therefore, the addition of deregulated MYC in this *in vivo* model system transforms cells into IDC in an MBII-dependent manner. Thus, we are able to evaluate MYC-driven tumour progression (10A.PM) against a non-transformed control (10A.PE) *in vivo*, as well as being able to measure tumour volume and evaluate tumour pathology: a rich data set that, to the best of our knowledge, uniquely distinguishes this model from previous solid human MYC-driven cancer models.

### 10A.PM tumours have MYC and invasive ductal carcinoma expression signatures

**Fig. 3.**
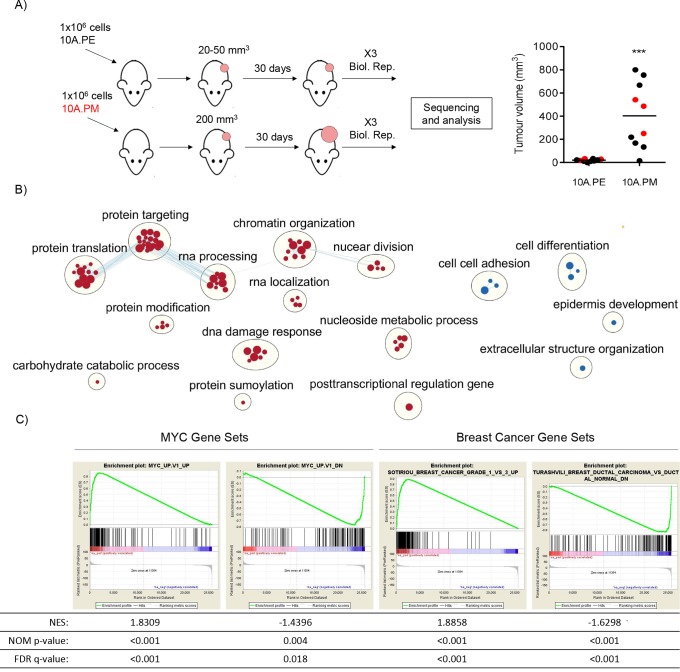
**10A.PM tumours have MYC and invasive breast cancer expression signatures.** (A) Female NOD-SCID mice were injected with 10A.PE or 10A.PM cells and allowed to form tumours (left). For RNA-seq analysis, 10A.PE growths (*n*=3) and actively growing10A.PM tumours (*n*=3) were harvested (right, indicated in red). ***P≤0.001, unpaired, two-tailed *t*-test. (B) Changes in gene expression, relative to 10A.PE xenografts, were ranked and tested for enrichment in GO Biological Process gene sets. The 10A.PM upregulated or downregulated genes that were significantly enriched in a GO Biological Process gene set are represented as red or blue circles, respectively (all data are provided in Table S2). Edges represent the number of overlapping genes within each gene set and node size represents the number of genes within a single gene set. Finally, clusters of gene sets and annotations were created using clusterMaker2 and AutoAnnotate Cytoscape applications. Data has been filtered (for visualisation) to show highest/lowest enriched GO processes (Reimand et al., 2019). (C) The gene expression data were analyzed by GSEA with MYC and IDC signature data sets. Gene sets used are labelled on the top of each enrichment plot (all data is provided in Table S2). NES, normalised enrichment score (the enrichment score for the gene set after it has been normalised across analysed gene sets). NOM *P*-value, the statistical significance of the enrichment score. The nominal *P*-value is not adjusted for gene set size or multiple hypothesis testing and, therefore, is of limited use in comparing gene sets. FDR *q*-value, false discovery rate (i.e. the estimated probability that the normalised enrichment score represents a false-positive finding).

Our histological analysis demonstrates that the *in vivo* 10A.PE and 10A.PM isogenic xenograft pair represents normal breast acini and IDC, respectively. We took this opportunity to identify an *in vivo* MYC-dependent transformation expression data set. We re-established 10A.PE and 10A.PM xenografts and isolated RNA from three biological replicates for RNA-sequencing (RNA-seq) (Fig. 3A). Contaminating mouse RNA was removed using Xenome (Conway et al., 2012), leaving ∼5000 significantly differentially expressed genes (PPDE ≥0.95, Table S1). A subset of upregulated and downregulated genes were validated through reverse transcription quantitative PCR (RT-qPCR) using human-specific primers and the 2(−ddC(T)) method (Fig. S1F) (Livak and Schmittgen, 2001). Following RNA-seq, we performed gene set enrichment analysis (GSEA) (Reimand et al., 2019; Subramanian et al., 2005) on ranked gene list expression data to generate a gene ontology (GO) map of upregulated and downregulated biological functions in 10A.PM tumours (Fig. 3B, Table S2). Upregulated biological functions included protein translation, chromatin organisation and RNA processing. Interestingly, downregulated processes included cell-cell adhesion and cell differentiation, reflective of aggressive breast cancer. Additionally, we performed GSEA to compare our observed differentially expressed genes with well-established MYC-driven and breast cancer gene sets (Fig. 3C, Table S2) (Liberzon et al., 2011). The MYC_UP.V1_UP gene set was significantly enriched in the upregulated portion of our xenograft gene expression list, whereas the MYC_UP.V1_DN gene list was significantly enriched in the downregulated portion of our xenograft gene expression list. These data show that the expression of known upregulated and downregulated MYC target genes is also observed in the 10A.PM model. Additionally, we observed significant and positive normalised enrichment scores (NES) with IDC gene sets (Fig. 3C, Table S2), indicating that these gene sets were identified in the upregulated portion of our xenograft gene expression list. These data reinforce the idea that our MYC-driven breast cancer model accurately recapitulates the activated and downregulated signalling pathways associated with MYC deregulation and IDC observed in human disease.

### Constitutive expression of deregulated MYC is required for tumour growth

**Fig. 4.**
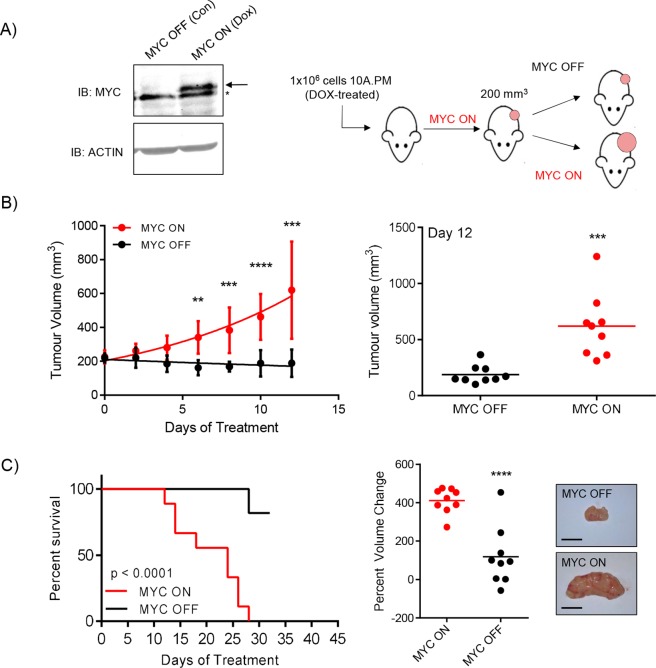
**Constitutive expression of deregulated MYC is required for tumour growth.** (A) With the addition of 1 μg/ml doxycycline (DOX) *in vitro*, ectopic MYC is expressed (MYC ON). The arrow indicates ectopic MYC; the asterisk indicates endogenous MYC. A total of 1×10^6^ cells were injected into female NOD-SCID mice (*n*=18) and given DOX-treated water (100 μg/ml) to induce MYC expression. Tumours were allowed to grow until 200 mm^3^, at which point the animals were randomised into MYC ON (*n*=9) or MYC OFF (*n*=9) groups. (B) Individual mean values from all measured tumours (nine per group) during the initial 12 day treatment period are shown (left) and individual tumour volumes from day 12 (final day with all animals present) are also shown (right); **P≤0.01, ***P≤0.001, ****P≤0.0001, unpaired, two-tailed *t*-test. Error bars represent s.d. (C) Left, tumours were measured until the mice reached a humane end point of 1000 mm^3^ or until they had received treatment for 1 month. Time to humane end point was plotted as percentage survival; ****P≤0.0001, log-rank test. Middle, percent volume changes for each tumour after the entire course of treatment are reported. ****P≤0.0001, unpaired, two-tailed *t*-test. Right, representative images are shown. Scale bars: 1 cm.

Introducing deregulated MYC into MCF10A cells expressing PIK3CA^H1047R^ transforms these cells *in vivo*; however, it is not clear whether deregulated MYC is necessary to sustain tumour growth. To address this, we generated cell lines with doxycycline (DOX)-inducible MYC to regulate ectopic expression (Fig. 4A, left). Cells were treated with DOX to induce ectopic MYC expression and injected subcutaneously into NOD-SCID mice, which were given supplemented DOX-treated drinking water. Once tumours reached 200 mm^3^, mice were randomly assigned to ‘MYC ON’ or ‘MYC OFF’ groups, the latter being removed from DOX-supplemented drinking water (Fig. 4A, right). Over 12 days of treatment, MYC ON tumours continued to grow and MYC OFF tumours slightly declined (Fig. 4B, left). MYC ON tumours had a doubling time of 7-8 days during this 12 day treatment period, whereas MYC OFF tumours, on average, were shrinking at a negative doubling rate of approximately 39 days. After 12 days of treatment, the first MYC ON tumour reached humane end point (Fig. 4B, right). Treatment was continued for 31 days and the overall time to humane end point for MYC ON tumours was significantly shorter than for MYC OFF tumours (Fig. 4C, left). In addition, we calculated the percent volume change for every tumour by comparing the final volume measurements (either at humane end point or 31 days of treatment) to the initial tumour volumes (approximately 200 mm^3^) and observed a significant difference between MYC ON and MYC OFF groups (Fig. 4C, right). These data demonstrate that this model is both driven by, and addicted to, deregulated MYC in a reproducible and quantitative manner. Furthermore, we show that targeting the deregulated fraction of MYC is sufficient to inhibit tumour growth, as endogenous MYC expression is not affected by DOX treatment (Fig. 4A). Therefore, the observed phenotypes, signalling pathways and vulnerabilities that arise *in vivo* are a consequence of the regulatable and ectopic allele of MYC and, importantly, are not due to permanent genomic or microenvironmental changes following deregulated MYC-driven transformation.

The isogenic MCF10A panel presented here offers unique advantages and is complementary to current MYC-dependent models of transformation (Fig. S1G) (Boxer et al., 2004; Wang et al., 2011; Zhao et al., 2003). Interestingly, the observed MYC-driven phenotype was exclusive to the *in vivo* xenograft assay. This observation highlights the importance of identifying a relevant context when studying MYC-dependent transformation, as our *in vitro* studies demonstrated little oncogenic MYC activity towards proliferation or *in vitro* acini transformation and was quite unable to predict the striking *in vivo* response. Moreover, studying cancer biology using *in vivo* models most accurately reflects human disease, and to some extent incorporates the contribution of tumour microenvironment and cellular stress. The *in vivo* context is therefore ideal for studying deregulated MYC and for identifying MYC-dependent vulnerabilities, as the relevant stress-signalling pathways will be active.

We have demonstrated that this model can recapitulate human breast cancer at both the molecular and pathological level, and is transformed by deregulated MYC. We anticipate that this model will be a useful tool for identifying the context-dependent functions and vulnerabilities of MYC-driven cancers, which are more difficult to discover using traditional *in vitro* or murine models*.* Additionally, this system is reproducible and scalable: ideal characteristics for the identification and validation of anti-MYC therapies. Indeed, we have recently demonstrated the utility of this model to interrogate MYC mutants for their role in transformation (Kalkat et al., 2018). We anticipate that this system will be able to predict the efficacy of novel therapeutic drugs targeting MYC and will identify novel vulnerabilities of MYC-dependent breast cancers.

## MATERIALS AND METHODS

### Cell culture

MCF10A epithelial cells were a kind gift of Dr Senthil Muthuswamy and were cultured in MCF10A culture media, as previously described (Debnath et al., 2003). All cell lines were routinely monitored for mycoplasma contamination and tested negative; *in vitro* experiments were conducted within five passages from selection. Cell culture images were taken with a 32× (Ph) objective on an AxioObserver microscope (Zeiss).

### Lentiviral vectors and cell-line transduction

cMYC and PIK3CA^H1047R^ (cloned from Addgene plasmid #12524) alleles were first cloned into pENTR4 no ccDB (686-1) (Addgene plasmid #17424) and subsequently shuttled into pLenti CMV/TO Puro DEST (670-1) (Addgene plasmid #17293) and pLenti CMV Hygro DEST (w117-1) (Addgene plasmid #17454), respectively (Campeau et al., 2009). Tetracycline-inducible cells were generated using pLenti CMV TetR Blast (716-1) (Addgene plasmid #17492). Lentiviral particles were generated using HEK293Tv (kind gift from Sam Benchimol) and MCF10A cell lines were transduced at the same time and with an equivalent viral titre.

### Antibodies

Immunoblotting was performed against pAKT^S473^ (Cat# 4051, Cell Signaling Technology), total AKT (Cat# 4685, Cell Signaling Technology), MYC (9e10, homemade, 1:1000 dilution), actin (Cat# A2066, Sigma), fibronectin (Cat# Ab32419, Abcam), vimentin (Cat# 5741, Cell Signaling Technology) and tubulin (Cat# CP06, Millipore). Antibody dilutions were used according to manufacturer's recommendations.

### EdU incorporation assay

The MCF10A isogenic panel was grown for 24 h in fully supplemented media and allowed to reach 50% confluency. At 50% confluency, cells were treated with 5 ethynyl-2′-deoxyuridine (EdU) for 4 h, processed according to manufacturer's instructions (Click-iT EdU Alexa Fluor 488 Flow Cytometry Assay Kit, Cat# C10420, Life Technologies) and analysed for EdU positivity using the LSR II flow cytometer (BD Biosciences) and FlowJo v10 software.

### Three-dimensional Matrigel morphogenesis assay

Cells were cultured for 12 days on Matrigel (Cat# 354230, Corning) in specialised MCF10A media for three-dimensional cultures, as previously described (Debnath et al., 2003). Images were acquired on day 12 using a FLUAR 5×/0.25 NA lens on an AxioObserver microscope (Zeiss). Cell-line groups were blinded and quantified on the basis of a qualitative assessment of acinar circularity and size. Small round acini were considered normal; large and disorganised acini were considered transformed. One-way ANOVA with Bonferroni post-test for multiple testing was used for analysis.

### Xenografts

A total of 1×10^6^ cells were suspended in 50% Matrigel (Cat# 354262, Corning) to a final volume of 0.2 ml and injected subcutaneously into the flanks of six- to eight-week old female NOD-SCID mice. For tetracycline-repressed cell lines, 100 μg/ml DOX (Sigma) was supplemented into the drinking water, prepared fresh twice a week. Animals were randomised to receive DOX or control water when tumours reached 200 mm^3^. The end point for all experiments was determined when tumours exceeded 1000 mm^3^ or if otherwise stated. Percent volume change was calculated for each tumour using the following formula: 100 × [(V_end_ − V_start_)/V_start_], where V_end_ is the final volume of each tumour at end-point and V_start_ is the initial volume when DOX-treatment began. Animal oversight was performed by the Animal Resources Centre (ARC) affiliated with the University Health Network (UHN).

### EMT analysis

Recombinant human transforming growth factor β (TGF-β) was purchased from PeproTech (Rocky Hill, NJ, USA) and reconstituted according to the manufacturer's instructions. The isogenic MCF10A panel presented in Fig. 1A was cultured in vehicle control or 5 ng/ml TGF-β for 72 h before harvest.

### Immunohistochemistry

Extracted tumours were fixed in 10% buffered formalin (Sigma) for 24-48 h at room temperature and then stored in 70% ethanol at 4°C. Samples were then paraffin-embedded, sectioned at 4 μm thickness and stained with haematoxylin and eosin (H&E). Ki67 (NB110-90592, Novus), TUNEL (homemade by the Pathology Research Program Laboratory), ER (ab80922, Abcam), PR (NCL-PR-312, Leica), HER2 (RM9103, Thermo Fisher Scientific), CK5 (NCL-LCK5, Leica), EGFR (28-0005, Invitrogen), p63 (VP-P960, Vector) and SMA (M0851, Dako) were used to further analyse tumour sections. All immunohistochemistry was performed as a service by the Pathology Research Program Laboratory (University Health Network, Toronto, Canada).

### RT-qPCR

The cDNA was synthesised using SuperScript III as per the manufacturer's protocol (Invitrogen). The mRNA expression was analysed by RT-qPCR using primers listed in Fig. S1H and normalised to the housekeeping gene TBP using SYBR Green (Thermo Fisher Scientific).

### RNA sequence analysis

Tumours were extracted and immediately flash-frozen. RNA from xenograft tissue was harvested using the RNeasy Plus Universal Kit (Cat# 73404, Qiagen) following the manufacturer's protocol. RNA was sent to the Princess Margaret Genomics Centre and sequenced using the Illumina NextSeq 500 for an average of 40 M reads per xenograft growth. Fastq files were filtered using Xenome in order to filter out mouse contamination. Only reads mapping exclusively to the mouse reference were filtered out. The remaining reads were mapped to the human reference genome (hg19) and read counts estimated using STAR. Expressed genes were defined as requiring at least 10 reads in at least two samples. Read counts were upper quantile normalised. Finally, EBSeq was used to test for differential expression between experimental conditions (Leng et al., 2013). Genes were determined to be differentially expressed given a false discovery rate (FDR) threshold of 0.05. Raw sequencing files can be found on GEO under accession number GSE130513.

### Gene ontology and gene set enrichment analysis

All available gene expression data between 10A.PE and 10A.PM were first ranked [GSEA Rank=−log(PPEE)×PostFC] and then used for GSEA with GO Biological Process gene sets using GSEA software (https://cytoscape.org/) following the protocol outlined in Reimand et al. (2019). Gene sets included the entire ‘GO gene set’ collection and selected MYC and breast cancer gene sets (Table S2). Significantly upregulated or downregulated GO processes were then used to generate a cytoscape map. Groups visualised have been filtered to NES=1.75-2.163 or −1.716-0. We acknowledge our use of the gene set enrichment analysis, GSEA software and Molecular Signature Database (MSigDB) (Subramanian et al., 2005).

## Supplementary Material

Supplementary information
