# Supplementary material for: Modelling the MYC-driven normal-to-tumour switch in breast cancer
Source: Dis Model Mech. 2019 Jul 26;12(7):dmm038083. doi: 10.1242/dmm.038083 (PMC6679384; doi:10.1242/dmm.038083)
Supplement: Supplementary information [file dmm-12-038083-s1.pdf]

A)

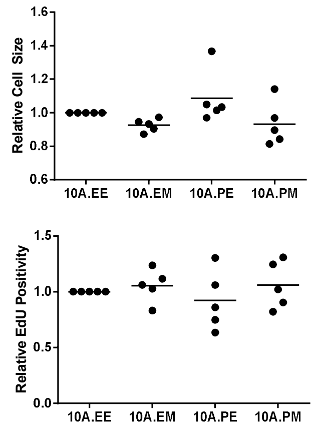

B)

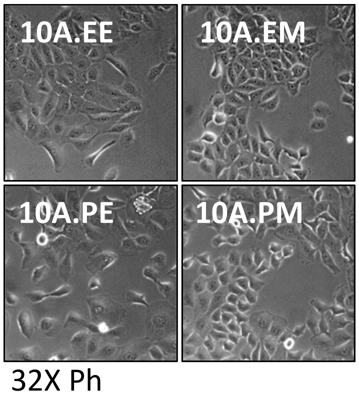

C)

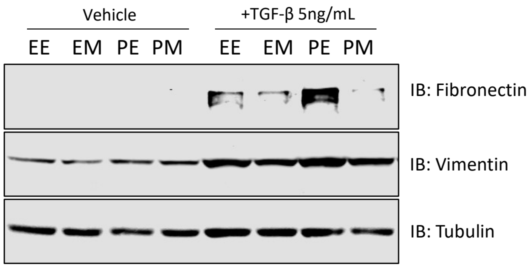

D)

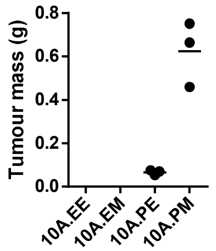

Xenograft Take Rate	
10A.EE	0/3
10A.EM	0/3
10A.PE	3/3
10A.PM	3/3

E)

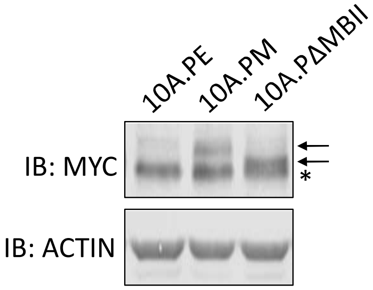

F)

GENE	10A.PE (LOG(2^{-ddCT}))	10A.PM (LOG(2^{-ddCT}))
MYC	0 (-0.19 - 0.19)	1.39 (1.29 - 1.50)
ODC1	0 (-0.67 - 0.67)	0.85 (0.78 - 0.93)
CDK4	0 (-0.57 - 0.57)	0.58 (0.50 - 0.65)
ENPP2	0 (-0.31 - 0.31)	-3.00 (-3.54 - -2.47)
CYBRD1	0 (-0.33 - 0.33)	-1.18 (-1.34 - -1.01)
HES1	0 (-0.23 - 0.23)	-0.36 (-0.59 - -0.12)

G)

		MMTV-rtTA/TetO-MYC	Breast Cell Line	MCF10A.PM Isogenic model
	Species of origin:	Mouse	Human	Human
	MYC Deregulation:	Inducible	Constitutive	Constitutive/Inducible
	Other oncogenic mutations:	Yes	HTERT, PIK3CA, SV40 LT	PIK3CA^{H1047R}, CDKN2A^{del}
MYC-dependent transformation:	Soft agar:	nr	Yes	nr
	Organoids:	nr	nr	No
	In vivo:	Yes	No	Yes
	In vivo growth time:	5-6 months	nr	1-2 months
	Human pathology:	No (mouse)	nr	Yes
	Molecular Profiling *In vivo:*	Yes	No	Yes

H)

GENE	Forward Primer	Reverse Primer
MYC	5’AGGGTCAAGTTGGACAGTGTC	5’TGGTGCATTTTCGGTTGTGG
ODC1	5’CCTTTTGGAACGGGCGAAAG	5’CAAAAACACAGCGGGCATCA
CDK4	5’CTGACCGGGAGATCAAGGTA	5’GGCTTCAGATCTCGGTGAAC
ENPP2	5’CATGCCAGAGGAAGTTACCAG	5’AGACCCTTTTGTATGAAGCCG
CYBRD1	5’AGCACTTATGGGATTGACAGAG	5’CCGAACACCAGGATCAGAAG
HES1	5’CGGACATTCTGGAAATGACA	5’GTGCGCACCTCGGTATTAAC
TBP	5’TATAATCCCAAGCGGTTTGC	5’GCTGGAAAACCCAACTTCTG

Fig. S1 – Deregulated MYC transforms breast acini into invasive ductal carcinoma *in vivo.*

(A) EdU proliferation measurements for the isogenic MCF10A panel analyzed by FLOW and forward scatter was used to measure cell size. Mean values are shown, one-way ANOVA with Bonferroni post-test for multiple testing, 5 biological replicates.(B) The MCF10A isogenic panel was cultured in standard MCF10A media for 48 hours before imaging to monitor cell morphology. (C) EMT was monitored by measuring fibronectin and vimentin expression 72 hours after treatment with vehicle alone or TGF-B (positive control). (D) The MCF10A isogenic panel was injected into female NOD-SCID mice as previously described. Summary of tumor formation is presented, 3 animals per group. Total mass of each tumour is displayed. 10A.PM form large tumours, PE form small, but palpable masses whereas 10A.EE and 10A.EM cells do not lead to the formation of masses. (E) Ectopic MYC expression from cells injected into NOD-SCID mice. Arrows point to ectopic WT and ΔMBII protein. * indicates endogenous MYC. (F) qRT-PCR analysis from 10A.PE and 10A.PM tumors. Fold expression changes LOG(2^{-ddCT}) (confidence intervals contained in brackets) are summarized in table format with genes significantly upregulated (from RNA-seq) in red and downregulated in blue. (G) Summary table of common GEMM and breast cell line models in comparison to the MCF10A.PM isogenic model of MYC-dependent transformation. “nr” represents not reported. (H) Primers used in this study.

Disease Models & Mechanisms • Supplementary information

A)

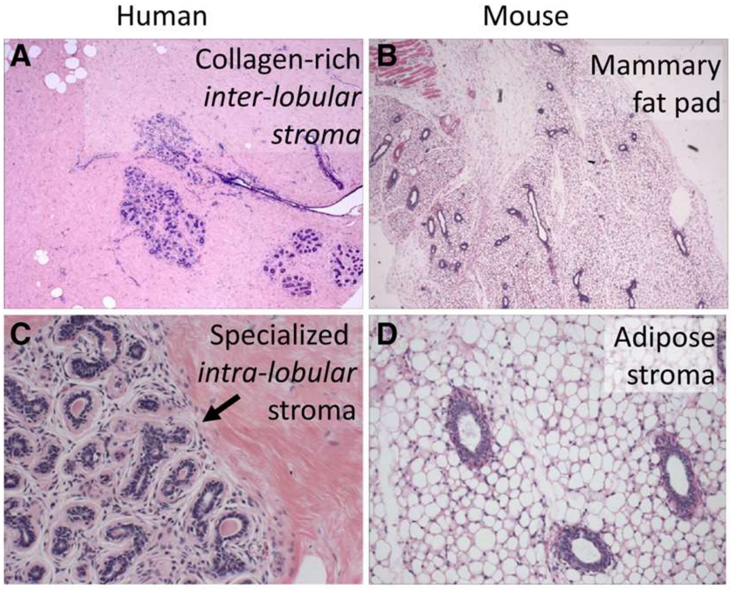

B)

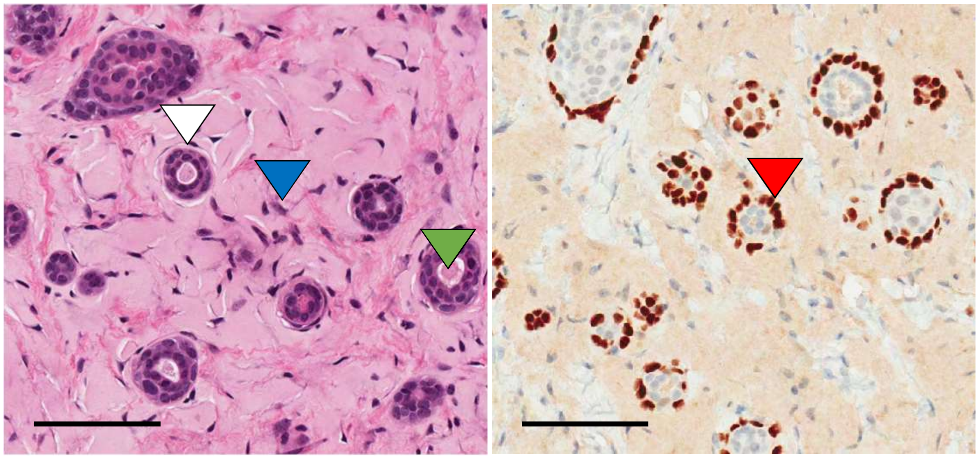

Arrow Colour	Feature
White	Bi-layered acinar structure
Green	Lumen
Blue	Stromal cells (mouse cells)
Red	Myoepithelial cells (p63)

Disease Models & Mechanisms • Supplementary information

Fig. S2 – Comparison between normal human breast histology and 10A.PE xenograft growths.

(A) Sample image demonstrating basic differences between human and mouse breast histology adapted from Dontu and Ince, 2015 (http://creativecommons.org/licenses/by/4.0/). 10A.PE growths are strikingly similar to human breast histology, despite forming sub-cutaneously in mice. **(B)** Example H&E (left) and α-p63 image (right) from a 10A.PE xenograft growth, highlighting features commonly observed in normal human breast tissue. Scale bars = 100µM

Table S1 – MYC driven 10A.PM xenograft differential expression analysis.

Differential expressed genes from three 10A.PM and 10A.PE xenograft tumours. Genes with a positive fold change are increased in 10A.PM relative to 10A.PE. Genes with a negative fold change are decreased in 10A.PM relative to 10A.PE. RealFC: real fold change, postFC: posterior fold change (LOG_2(RealFC)), PPEE: posterior probability of being equally expressed, PPDE: posterior probability of being differentially expressed, GSEA Ranking: -LOG(PPEE)*PostFC.

Click here to Download Table S1

Disease Models & Mechanisms • Supplementary information

Table S2 – Summary of Gene Set Enrichment Analysis.

Tab 1 – Summary compilation of all GO Biological Processes gene sets analyzed using GSEA. Size: Number of genes in the gene set after filtering out those genes not in the expression dataset, NES: normalized enrichment score (the enrichment score for the gene set after it has been normalized across analyzed gene sets), NOM p-value: the statistical significance of the enrichment score. The nominal p value is not adjusted for gene set size or multiple hypothesis testing; therefore, it is of limited use in comparing gene sets, FDR q-value: False discovery rate; that is, the estimated probability that the normalized enrichment score represents a false positive finding, Rank at max: The position in the ranked list at which the maximum enrichment score occurred. The more interesting gene sets achieve the maximum enrichment score near the top or bottom of the ranked list; that is, the rank at max is either very small or very large. **Tab 2** – Summary compilation of all MYC and breast cancer gene sets analyzed using GSEA. Size: Number of genes in the gene set after filtering out those genes not in the expression dataset, NES: normalized enrichment score (the enrichment score for the gene set after it has been normalized across analyzed gene sets), NOM p-value: the statistical significance of the enrichment score. The nominal p value is not adjusted for gene set size or multiple hypothesis testing; therefore, it is of limited use in comparing gene sets, FDR q-value: False discovery rate; that is, the estimated probability that the normalized enrichment score represents a false positive finding. Rank at max: The position in the ranked list at which the maximum enrichment score occurred. The more interesting gene sets achieve the maximum enrichment score near the top or bottom of the ranked list; that is, the rank at max is either very small or very large.

Click here to Download Table S2

Disease Models & Mechanisms • Supplementary information
